# Higher self-reported prevalence of hypertension among Moluccan-Dutch than among the general population of the Netherlands: results from a cross-sectional survey

**DOI:** 10.1186/1471-2458-14-1273

**Published:** 2014-12-15

**Authors:** Junus M van der Wal, Adee J Bodewes, Charles O Agyemang, Anton E Kunst

**Affiliations:** Department of Public Health, Academic Medical Centre, University of Amsterdam, Meibergdreef 9, 1105 AZ Amsterdam, the Netherlands

**Keywords:** Hypertension, the Netherlands, Ethnicity, Acculturation, Moluccans

## Abstract

**Background:**

Several studies in the Netherlands revealed ethnic disparities in hypertension prevalence, but none have focused on the Moluccan-Dutch, a migrant group from Indonesia that settled in the Netherlands in 1951. The Moluccan-Dutch are considered to be fairly well integrated in Dutch society. The aim of this study was to compare hypertension prevalence among the Moluccan-Dutch to the native Dutch and to explore the contribution of known risk factors.

**Methods:**

A health interview survey was conducted from August 2012 till March 2013 among nineteen Moluccan neighborhoods, resulting in the inclusion of 708 participants. The primary outcome variable was self-reported prevalence of hypertension. Explanatory variables were BMI, exercise, smoking, alcohol intake and mental health status. Data on the control group was extracted from the Dutch National Health Survey 2011, using a similar questionnaire. Differences in risk factor exposure were explored using Chi-square tests and the contribution of risk factors, separately and combined, was explored using multivariate logistic regression analysis.

**Results:**

Moluccan-Dutch showed higher odds for reporting hypertension when compared to native Dutch, after adjusting for age and level of education (OR = 1.38; 95% CI = 1.13-1.69) and additional risk factors (OR = 1.49; 95% CI = 1.19-1.88). A higher prevalence of hypertension was found in both Moluccan-Dutch men (26.4% vs. 16.7%; p < 0.001) and women (26.7% vs. 17.9%; p < 0.001), when compared to the control group. Not only middle-aged, but also young Moluccan-Dutch men showed higher prevalence of hypertension.

**Conclusion:**

The Moluccan-Dutch may be at increased risk for reporting hypertension. These results suggest that long-term stay over several generations does not necessarily result in similar levels of hypertension prevalence as the host population.

## Background

Within the Netherlands, cardiovascular disease (CVD) is the second most common cause of death [1]. In the last decades, a major research effort elucidated striking differences in cardiovascular health between different ethnic groups in high-income countries [2, 3]. Within the Netherlands, multiple studies revealed ethnic disparities in the prevalence of hypertension, a major risk factor for CVD [4, 5]. Immigrant groups in Amsterdam from Surinamese, African and South-Asian descent were found to have higher prevalence of hypertension than the European origin populations [6–8].

There is an absence of sufficient data on the Moluccan-Dutch, an ethnic minority group that has been in the Netherlands for multiple generations. In spring 1951, 12,500 Moluccans, former soldiers of the Royal Netherlands East Indies Army (KNIL) and their families, traveled from Indonesia to the Netherlands. Despite their intention to return to the Moluccan Islands, the majority settled in the Netherlands. No significant second migration wave occurred, resulting in a fairly static minority group [9]. A study in 2001 estimated that there were 45,000 people of Moluccan descent in the Netherlands [10]. The Moluccan-Dutch are considered to be fairly well integrated in Dutch society [11], but at the same time still adhere to some aspects of traditional Moluccan culture. Persistent features such as traditional cuisine, strong adherence to religion, separate community life, and cohesive family ties still influence everyday-life. Furthermore, local Moluccan dialects such as Ternatean- and Ambonese Malay are still being spoken in Moluccan neighborhoods in the Netherlands. Despite having adjusted to life in Dutch society after 60 years, Moluccans still show considerable awareness of and interest in their cultural heritage. Their place in Dutch society exemplifies a process of segmented assimilation [12, 13].

The fact that Moluccan-Dutch have been in the Netherlands much longer than any other ethnic minority group, but do not fully integrate in Dutch society, provides a unique opportunity to assess whether ethnic disparities in health could endure after considerable length of residence in the host country. This question is relevant, since multiple studies have suggested that the length of residence of an immigrant group will influence the health status of these people. Early studies in Japanese immigrants in the United States sparked the hypothesis that acculturation is a considerable factor of importance in ethnic disparities in coronary heart disease (CHD) [14]. Empirical evidence on length of residence, acculturation and cardiovascular health compiled since then shows an ambiguous relationship. Some studies give the impression that immigrant groups with lower incidence of CVD and risk factors converge to more unfavorable figures of the new host country [15, 16], whereas a higher incidence of CVD in first generation sometimes persists in the second generation [17, 18]. The relationship between length of stay and CVD is believed to be due mainly to a shifting risk profile [19, 20]. Acculturation is often proposed to be a leading mechanism in this shifting risk profile, especially in the context of diet [21] and other cardiovascular risk factors [16, 22, 23].

There is little empirical evidence on the health status of the Moluccan-Dutch. A study by Ho et al. revealed that first generation, but not second generation Indonesian immigrants, displayed a higher risk of dying from CVD than native Dutch [24]. Similarly, van Oeffelen et al. show that first generation Indonesian immigrants display a higher incidence of acute myocardial infarction when compared to native Dutch; the second generation Indonesian immigrants shows an inverted relationship [13]. Nonetheless, both of these studies focused on Indonesian immigrants as a whole, a major part of which come from outside the Moluccan Islands. One study that surveyed general practitioners in Moluccan-Dutch communities estimated the prevalence of hypertension and CVD to be significantly higher among the Moluccan-Dutch when compared to the native Dutch [25].

The main purpose of this study was to compare the prevalence of hypertension in the Moluccan-Dutch community with the general Dutch population, and to explore the influence of known risk factors for hypertension. Based on previous studies on Indonesian and Moluccan immigrants and studies on other ethnic minorities in the Netherlands, we expect the prevalence of hypertension to be higher among the Moluccan-Dutch than among the native Dutch. Such a higher prevalence would be consistent with the process of segmented assimilation, and it would suggest that elements of traditional Moluccan culture have a persistent effect on hypertension risk. On the other hand, if prevalence of hypertension would be similar or close to the levels of native Dutch, this would suggest that a process of long-term acculturation had been of dominant influence.

## Methods

We carried out a survey among nineteen Moluccan communities. The questionnaire consisted of seven parts: demographics and work, acculturation and language, general wellbeing, illness and disease, healthcare use, (voluntary) care giving, and lifestyle. Questions were based on the National Health Survey questionnaire of the Dutch Central Bureau of Statistics/Statistics Netherlands (CBS) [26]. Questions specific to the Moluccan culture, such as traditional Moluccan diet or medicinal practices, were added.

The questionnaire was sent to all Moluccan-Dutch people between the ages of 30 till 65 who resided in nineteen Moluccan neighborhoods throughout the Netherlands. The neighborhoods were selected to be representative of all sixty Moluccan neighborhoods in terms of size, degree of urbanization, region, and dominant religion. Addresses were provided by local Moluccan organizations. Respondents had the option to complete the questionnaire on paper or online, or to be interviewed personally during a home visit. Non-respondents received a reminder two weeks after the questionnaire was sent.

Data on the native Dutch control group was extracted from the National Health Survey 2011 conducted by Statistics Netherlands (CBS). This survey was carried out among a random sample of the Dutch population drawn from municipal registries. For this analysis, we selected respondents between 30 and 65 with self-reported Dutch origin [27].

The primary outcome variable was prevalence of hypertension, based on the acknowledgment of hypertension existence in the last 12 months in a question exploring current chronic diseases. Risk factors were BMI, exercise, smoking, drinking habits and mental health status. BMI was calculated using self-reported height and bodyweight. Exercise was measured by number of strenuous activities performed each week, such as cycling, gardening or carrying heavy load, as reported in the lifestyle section in the questionnaire. Smoking habits and alcohol consumption were determined based on self-reported number of tobacco products smoked daily and number of alcoholic beverages consumed weekly. Mental health status was determined using the MHI-5 scale [28].

### Statistical analysis

Differences in the prevalence of hypertension were explored using a Chi-square test. The contribution of each risk factor to the difference in hypertension prevalence between Moluccan-Dutch and native Dutch was determined using multivariate logistic regression analysis. All regression models are stratified by sex and controlled for age and level of education. The contribution of other risk factors was assessed for each risk factor individually, by adding the risk factor to the basic model. For smoking and alcohol, we included ordinal variables with non-smoking and moderate alcohol intake as the reference group. BMI, exercise and mental health were included in similar ways. A p-value ≤0.05 was considered to indicate statistical significance. Data were analyzed using PASW statistics for Windows (IBM Corp. Released 2010. IBM SPSS Statistics for Windows, Version 19.0. Armonk, NY: IBM Corp.).

### Ethical approval

The Medical Ethical Committee (METC) of the Academic Medical Center granted an exemption from requiring ethnical approval, because the Medical Research Involving Human Subjects Act (WMO), which governs medical research in the Netherlands, does not apply to this study.

## Results

### Characteristics of the study population

In total 2935 questionnaires were sent and 755 persons returned a questionnaire. We excluded participants who were evidently not of Moluccan descent, who fell outside the age criteria for this study, or who failed to correctly complete most parts of the questionnaire, especially the question regarding hypertension. In the end 708 participants were included for analysis.

Basic demographic characteristics are presented in Table 1. There were significant differences between Moluccan-Dutch and native Dutch in all basic demographic characteristics. Compared to Moluccan-Dutch, the sample of native Dutch inhabitants was younger, higher educated and more likely to be cohabitating.

**Table 1:** **Basic demographic characteristics of Moluccan-Dutch study population and native Dutch control group**

	Men (n = 1962)			Women (n = 2163)		
	Moluccan- Dutch (n = 341)	Native Dutch (n = 1621)	p	Moluccan-Dutch (n = 367)	Native Dutch (n = 1796)	p
**Age (years)**	56 (51 – 60)	51 (42 – 59)		55 (50 – 60)	50 (41 – 58)	
**Age categories (%)**			<0.001			<0.001
30 – 39	6.2	19.4		8.4	19.7	
40 – 49	14.4	26.0		16.1	29.1	
50 – 59	50.1	32.6		49.6	31.8	
60 – 65	29.3	22.0		25.9	19.4	
**Level of education (%)**			<0.001			<0.001
Primary	2.1	7.6		4.4	10.2	
Secondary	41.3	22.6		46.6	25.0	
Secondary vocational	33.4	30.1		32.2	30.0	
Higher	20.2	36.6		13.6	31.0	
Missing	2.9	3.1		3.3	3.8	
**Cohabitation (%)**			0.017			<0.001
Yes	76.8	82.2		61.9	81.6	
No	23.2	17.6		37.6	18.0	
Missing		0.2		0.5	0.4	

Age is displayed in median and interquartile ranges. Value p is calculated by Chi-square test.

Prevalence rates of risk factors for CVD are presented in Table 2. The Moluccan-Dutch men had somewhat higher prevalence of overweight than native Dutch (p = 0.064). There was no statistically significant difference in the proportion of current smokers, though native Dutch women seemed to smoke more than Moluccan-Dutch women. Both Moluccan-Dutch men and women consumed alcohol less often than their native Dutch counterparts (p < 0.001). The Moluccan-Dutch more often reported lower scores on the MHI-5 scale than the native Dutch, both in men (p < 0.001) and women (p < 0.001). However, in both groups only a small proportion of the respondents had a MHI-5 score below 60, which denotes poor mental health in the general Dutch population according to the Dutch Central Bureau of Statistics [26]. Prevalence of other risk factors showed no significant difference between the Moluccan-Dutch and native Dutch.

**Table 2:** **Exposure to risk factors in the Moluccan-Dutch study population and native Dutch control group**

	Men			Women		
	Moluccan-Dutch (n = 341)	Native Dutch (n = 1621)	p	Moluccan-Dutch (n = 367)	Native Dutch (n = 1796)	P
**BMI categories (%)**			0.064			0.183
Underweight (<18)	0.3	0.4		1.6	1.3	
Normal (18–25)	33.4	41.5		46.9	53.0	
Overweight (25–30)	49.0	46.4		35.4	30.5	
Obese	15.0	11.0		12.3	12.8	
Missing	2.3	0.7		3.8	2.5	
**Different exercise activities on a weekly basis (%)**			0.162			<0.001
0 – 2	20.8	24.9		19.9	21.2	
3 – 5	56.0	54.7		66.8	57.2	
6 – 8	23.5	20.4		13.3	21.7	
**Smoking (%)**			<0.001			<0.001
Never smoked	24.1	36.2		31.3	38.4	
Past smoker	49.7	41.1		42.5	40.4	
Current smoker						
*1 – 10 per day*	13.5	10.4		19.9	11.5	
*11 – 20 per day*	8.8	9.4		4.6	8.1	
*>20 per day*	1.8	2.8		1.6	1.7	
Missing	0.2	0		0	0	
**Alcohol consumptions per week (%)**			<0.001			<0.001
Abstainers	34.0	11.2		55.6	24.0	
Habitual (1 – 14)	58.1	66.7		41.1	66.8	
Often (15 – 28)	5.9	16.9		0.3	6.0	
Excessive (>28)	1.5	3.4		0.0	0.6	
Missing	0.6	1.8		3.0	2.6	
**People engaging in binge drinking episodes (%)**			<0.001			<0.001
Daily	1.5	2.5		0.3	0.4	
Weekly	7.3	13.6		1.1	3.1	
Monthly	38.7	32.7		22.3	11.4	
Never	29.3	39.4		39.8	61.3	
Missing	23.2	11.8		36.5	23.8	
**Mental health status (%)**			<0.001			0.008
MHI-5 > 80	51.9	64.3		44.1	53.4	
MHI-5 60 - 80	37.8	24.8		38.7	31.7	
MHI-5 < 60	8.2	9.5		12.8	11.9	
Missing	2.1	1.5		4.4	3.0	

MHI-5 = value on the Mental Health Inventory 5-scale. All p-values represent the outcome of a Chi-square test, testing for even distribution between different categories.

### Ethnic differences in hypertension

Differences in the prevalence of hypertension are shown in Table 3. There was a significant difference between Moluccan-Dutch and native Dutch, in both men (26.4% vs. 16.7% resp.; p < 0.001) and women (26.7% vs. 17.9% resp.; p < 0.001). Difference in hypertension prevalence was observed in most age categories, including those younger than 50 years.

**Table 3:** **Prevalence of hypertension in the Moluccan-Dutch study population and native Dutch control group**

	Men			Women		
	Moluccan-Dutch (n = 341)	General Dutch pop. (n = 1621)	p	Moluccan-Dutch (n = 367)	General Dutch pop. (n = 1769)	p
**Hypertension by age categories (%)**			0.008^1^			0.122^1^
30 – 39	9.5	5.1	0.381	6.5	5.1	0.745
40 – 49	16.3	10.9	0.262	20.3	11.7	0.056
50 – 59	31.0	20.5	0.004	25.3	24.5	0.837
60 – 65	27.0	29.4	0.638	40.0	27.8	0.022
**Total group**	26.4	16.7	<0.001^2^	26.7	17.9	<0.001^2^

1: p-value of the Chi-square statistic for distribution between Moluccan-Dutch and native Dutch.

2: p-value of the Chi-square statistic for difference in hypertension prevalence between Moluccan-Dutch and native Dutch.

Odds ratios for reporting hypertension by a Moluccan-Dutch person, when compared to a native Dutch, are presented in Table 4. Higher odds were found for both Moluccan-Dutch men (OR = 1.38 95% CI = 1.04-1.83; p = 0.011) and women (OR = 1.35; 95% CI = 1.03-1.77; p = 0.034). The high odds persisted after adjusting for age, level of education and risk factor exposure. The OR only diminished substantially after adjusting for alcohol intake in the female group (from 1.313 to 1.225).

**Table 4:** **Binary logistic regression analysis for hypertension prevalence among Moluccan-Dutch**

	Men		Woman		Total	
	OR	95% CI*	OR	95% CI*	OR	95% CI*
**When adjusted for**						
BD	1.458	1.089 – 1.952	1.319	0.997 – 1.745	1.384	1.130 - 1.694
BD + BMI	1.430	1.056 – 1.936	1.400	1.045 – 1.875	1.414	1.146 - 1.744
BD + Smoking	1.447	1.079 – 1.941	1.360	1.024 – 1.804	1.395	1.138 - 1.710
BD + Alcohol	1.455	1.075 – 1.970	1.225	0.913 – 1.644	1.324	1.073 – 1.635
BD + Alcohol (incl. binge drinking)	1.505	1.107 – 2.047	1.237	0.911 – 1.679	1.364	1.099 – 1.694
BD + Exercise	1.484	1.107 – 1.990	1.330	1.003 – 1.763	1.393	1.137 –1.706
BD + Mental health	1.488	1.102 – 2.007	1.308	0.980 – 1.746	1.389	1.129 – 1.709
All risk factors	1.492	1.079 – 2.064	1.395	1.012 – 1.924	1.420	1.133 – 1.780
All risk factors (incl. binge drinking)	1.573	1.132 – 2.185	1.413	1.013 – 1.971	1.494	1.185 – 1.883

BD = basic demographics (age and level of education); OR = odds ratio; CI = confidence interval; when controlling for additional risk factors, ordinal variables were used. *p < 0.05.

## Discussion

Our results are suggestive of a higher prevalence of hypertension in Moluccan-Dutch men and women, when compared to the native Dutch population. Notably, not only middle-aged, but also young Moluccan-Dutch men in our study population showed a higher prevalence of hypertension than their native Dutch counterparts. Even though exposure to various life style risk factors differed between the Moluccan- and native Dutch, these risk factors did not substantially influence the odds for the prevalence of hypertension among the Moluccan-Dutch.

This study has a number of limitations that should be evaluated. First of all, occurrence of hypertension was determined using self-report instead of measurements. This is likely to result in an underestimation of hypertension prevalence in both the Moluccan-Dutch and the control group. Further analyses of the survey data suggest that the Moluccan-Dutch in our study population are less likely to visit a physician than native Dutch (results not shown). This might result in a greater underestimation of hypertension among Moluccan-Dutch as compared to native Dutch, and thus underestimation of the differences between the two groups.

The response rate of 25.7% in the Moluccan-Dutch survey is low as compared to the response rate in the Dutch National Health Survey [27] between 60-65%. As a result, bias related to non-response is likely to have greater effect among Moluccan-Dutch. The direction of bias is uncertain, as it is unknown whether having hypertension increases the odds of completing and returning a health questionnaire. Perhaps Moluccans with disease were more likely to respond. However, this is not supported by the relatively low levels of health care utilization observed in our sample.

As this study was aimed to compare Moluccan-Dutch to the native Dutch, we did not make comparisons among Moluccan-Dutch participants. Yet, it might have been of interest to assess whether the prevalence of hypertension is related to the degree to which participants felt integrated into Dutch society. There were some variables in the survey that could potentially serve as measures of integration, such as Dutch language proficiency or ethnic identity. Language proficiency was high especially among younger Moluccan-Dutch, and slightly less among older Moluccans. Only 8% of the Moluccan-Dutch identified themselves as Dutch; the great majority (87,7%) identified themselves as Moluccan. Yet, we found no consistent or statistically significant correlation between Dutch language proficiency or ethnic identity and the likelihood of reporting hypertension.

Our results support previous suspicion among general practitioners that Moluccan-Dutch patient are at greater risk of having hypertension than native Dutch [25]. In addition, when placing our results in international perspective, the prevalence of hypertension is suggested to be higher in Indonesia as compared to the Netherland. Statistics Netherlands (CBS) reported a prevalence of hypertension of 16% in 2011 based on a cross-sectional survey among Dutch citizens of all ages. According to the World Health Organization (WHO), the prevalence of hypertension in Indonesia in 2008 was 32.5% among males and 29.3% among females [29]. It seems that the hypertension prevalence in our study group of Moluccan-Dutch is in-between Dutch and Indonesian values.

A higher prevalence of hypertension among the Moluccan Dutch, including the youngest age groups, might be explained by both social and biomedical factors. Despite the general opinion that the Moluccan-Dutch are well integrated in Dutch society, there are signs that their integration had only been partial. Moluccan Dutch rather exemplify a process of segmented assimilation. Moluccan-Dutch of the third generation show a high interest in their cultural heritage, even more so than the second generation [12]. Some aspects of traditional Moluccan culture are likely to influence their cardiovascular risk profile. For example, regular consumption of traditional Moluccan dishes, which often contain excessive amounts of salt, sugar or fat, might influence the risk of hypertension.

Biomedical factors are also likely to contribute to the difference in hypertension prevalence between Moluccan-Dutch and native Dutch in our study. Nguyen et al. showed that Indonesians, when compared to Vietnamese or Chinese, shown increasing odds for developing hypertension at lower BMI. This is probably attributable to differences in body fat volume at similar BMI levels [30]. One study documented differences in body composition between people of Indonesian and Dutch descent [31]. This physiological trait might contribute to the Moluccan-Dutch differences in prevalence of hypertension despite the small or non-existent differences in overweight.

The less optimal mental health status of the Moluccan-Dutch may, at least in theory, partly explain the difference in hypertension prevalence. A growing body of evidence supports a longstanding hypothesis that psychosocial stress, especially chronic stress and depression, influences occurrence of hypertension [32, 33]. Depressive symptoms are associated with a higher exposure to risk factors for CVD [34]. However, in our study, controlling for mental health barely altered the difference in the occurrence of hypertension between the ethnic groups. Further investigation using more detailed measures of mental health might provide more insight into the increased hypertension risk of Moluccan-Dutch.

We found that the higher prevalence of hypertension of Moluccan-Dutch women was in part explained by controlling for alcohol consumption. However, Moluccan-Dutch women consumed substantially less alcoholic beverages than the native Dutch women. It is possible that they might not benefit from proposed positive effects of moderate alcohol consumption in women, such as decreased occurrence of hypertension and metabolic biomarkers associated with CVD [35–38]. A speculative explanation might be that Moluccan-Dutch women are more likely to abstain from alcohol after being diagnosed with hypertension, a standard health advice in Dutch general practitioners offices [39].

Factors that fall outside the scope of our survey, such as racial discrimination [40] or genetics, might partly explain the differences found [41–43]. However, current research does not provide enough evidence to determine the contribution of these factors to the difference in hypertension prevalence observed in our study.

## Conclusion

The study limitations call for verification of our results by research using objective measurement of hypertension. Even so, our results could prompt healthcare providers in Moluccan-Dutch communities to aim at improving quality of care, especially cardiovascular risk management. A more thorough hypertension screening of Moluccan-Dutch patients may potentially lead to better cardiovascular health.

Furthermore, our study provides clues about the validity of the acculturation theory by suggesting that long-term stay over several generations does not necessarily result in similar levels of hypertension prevalence as the host population. Ongoing research is needed to clarify ethnic disparities in cardiovascular health and the mechanism underlying such disparities, particularly as we continue to unveil new minority groups potentially at risk for circulatory disease.

## Authors’ information

Junus M van der Wal is a medical student at the University of Amsterdam, currently in possession of a bachelor’s degree.

Adee J Bodewes (BSc) is staffed at the department of public health at the Academic Medical Center in Amsterdam, through her work for the Moluccans & Health project.

Dr. Charles O Agyemang (PhD) is a scientific staff member of the department of public health at the Academic Medical Center in Amsterdam and one of the institute’s principal investigators.

Prof. Dr. Anton E Kunst is a professor in social epidemiology at Academic Medical Center in Amsterdam and one of the institute’s principal investigators.
